# Comparison of Fungal Thermophilic and Mesophilic Catalase–Peroxidases for Their Antioxidative Properties

**DOI:** 10.3390/antiox12071382

**Published:** 2023-07-04

**Authors:** Andrej Poljovka, Miloš Musil, David Bednář, Katarína Chovanová, Vladena Bauerová-Hlinková, Jana Bellová, Lenka Kohútová, Peter Baráth, Marcel Zámocký

**Affiliations:** 1 Institute of Molecular Biology, Slovak Academy of Sciences, Dúbravská Cesta 21, 84551 Bratislava, Slovakia; andrej.poljovka@savba.sk (A.P.); vladena.bauerova@savba.sk (V.B.-H.); 2 Loschmidt Laboratories, Department of Experimental Biology and RECETOX, Faculty of Science, Masaryk University, 61137 Brno, Czech Republic; imusilm@fit.vutbr.cz (M.M.);; 3 International Clinical Research Centre, St. Anne’s University Hospital Brno, 65691 Brno, Czech Republic; 4 Department of Information Systems, Faculty of Information Technology, Brno University of Technology, 61200 Brno, Czech Republic; 5 Department of Glycobiology, Institute of Chemistry, Slovak Academy of Sciences, Dúbravská Cesta 9, 84538 Bratislava, Slovakiapeter.barath@savba.sk (P.B.); 6 Department of Inorganic Chemistry, Faculty of Natural Sciences, Comenius University in Bratislava, Mlynská Dolina, Ilkovičova 6, 84215 Bratislava, Slovakia

**Keywords:** peroxidase–catalase superfamily, heme catalase, bifunctional enzyme, reactive oxygen species, oxidative stress

## Abstract

Catalase–peroxidases (KatGs) are unique bifunctional oxidoreductases that contain heme in their active centers allowing both the peroxidatic and catalatic reaction modes. These originally bacterial enzymes are broadly distributed among various fungi allowing them to cope with reactive oxygen species present in the environment or inside the cells. We used various biophysical, biochemical, and bioinformatics methods to investigate differences between catalase–peroxidases originating in thermophilic and mesophilic fungi from different habitats. Our results indicate that the architecture of the active center with a specific post-translational modification is highly similar in mesophilic and thermophilic KatG and also the peroxidatic acitivity with ABTS, guaiacol, and L-DOPA. However, only the thermophilic variant CthedisKatG reveals increased manganese peroxidase activity at elevated temperatures. The catalatic activity releasing molecular oxygen is comparable between CthedisKatG and mesophilic MagKatG1 over a broad temperature range. Two constructed point mutations in the active center were performed selectively blocking the formation of described post-translational modification in the active center. They exhibited a total loss of catalatic activity and changes in the peroxidatic activity. Our results indicate the capacity of bifunctional heme enzymes in the variable reactivity for potential biotech applications.

## 1. Introduction

Catalases and peroxidases represent two key enzymes that act on reactive peroxide bonds in hydrogen peroxide and also in organic peroxides [1,2]. They are present in all aerobically metabolizing organisms. Catalases and peroxidases evolved in ancestral genomes independently as many diverse gene families, however, sharing many common features [3]. Their physiological function is manifold and diverse depending on their specific reaction mechanism. Peroxidases can only reduce H_2_O_2_ and concomitantly oxidize various substrates according to Equation (1)—where SH_2_ is substrate serving as an electron donor. Catalases can both reduce and oxidize this frequently occurring reactive molecule with signaling function according to Equation (2).

H_2_O_2_ + 2 SH_2_ → H_2_O + 2^.^SH (1)

2 H_2_O_2_ → 2 H_2_O + O_2_ (2)

Both these essential enzyme classes are systematically classified in RedoxiBase [4] in various families and superfamilies. Catalase–peroxidases (EC 1.11.1.21) represent unique bifunctional heme enzymes capable of both hydrogen peroxide dismutation and oxidation of a broad spectrum of mainly organic substrates with the help of simultaneous hydrogen peroxide reduction. It probably depends on the physiological conditions which type of reaction is preferred in a particular case. In older literature, they are abbreviated as KatGs [5,6], apparently derived from the primary annotation of corresponding genes in the *E. coli* genome and also in genomes of related bacteria. They all belong to the large peroxidase–catalase superfamily containing heme *b* as a prosthetic group. They are supposed to be at the ancestral roots of this complex gene family evolution [7]. KatGs were for a long time expected to be present only among bacteria and archaea but later they were also detected and purified among various fungi [6] and protists. Among certain eukaryotes, they occurred during evolution apparently after a horizontal (lateral) gene transfer event from prokaryotes [8]. The important fact is that after HGT, they retained the architecture of their active centers. This is obvious from the occurrence of the catalytic triad represented by highly conserved Arg, Trp, and His on the distal side of heme [9]. Further, an important post-translational modification occurs formed by covalent adduct between three conserved amino acids on the distal side of heme. This was detected first for cyanobacterial KatGs [10] but a comparable detection for abundant fungal KatGs was not demonstrated yet. Among fungi, KatGs are frequently present as two isoforms (intracellular and extracellular) mainly in phytopathogens [9]. In this contribution, we compare the behavior and structure of mesophilic and thermophilic counterparts of these unique fungal bifunctional oxidoreductases. Whereas mesophilic KatGs originate predominantly in numerous ascomycetous pathogens, rare thermophilic variants occur only among saprotrophic fungi. In this contribution, we try to find substantial differences that can contribute to increased thermostability of selected ascomycetous catalase–peroxidases with consequences for their possible biotech applications where thermostable enzymes with increased operational stability are advantageous. Our research in this contribution is focused on three important aspects: detection of an essential post-translational modification at the same protein location in thermophilic and mesophilic KatG, inspection of their whole protein molecules for potential differences that can contribute to increased thermostability, and comparison of their enzymatic activity and stability.

## 2. Materials and Methods

### 2.1. Cloning of Synthetic Genes for Thermostable and Mesophilic Catalase–Peroxidases

Synthetic CthediskatG gene with additional C-terminal His-tag was produced with codon optimization for *E. coli* expression at the company General Biosystems, Durham, NC, USA. It has, after translation, the same amino acid sequence as the naturally occurring catalase–peroxidase from the filamentous fungus *Chaetomium thermophilum* var. *dissitum* registered under RedoxiBase ID 14618 (GenBank: MF380422). It has a total length of 723 amino acids with an additional 6 C-terminally attached Histidine residues. This synthetic construct was cloned into vector pET21a using NotI and NdeI restriction sites. Clones for expression were selected and maintained on LB medium supplemented with Ampicillin (100 mg/mL). In a similar way, synthetic MagKatG1 gene with C-terminal His-tag with sequence identical with *Magnaporthe oryzae* katG deposited under RedoxiBase ID 2288 (Genbank: FD509783) containing 750 amino acids was cloned in vector pET21d and expressed under the same conditions.

### 2.2. Site-Directed Mutagenesis

To introduce point mutations in the cloned wild-type CthediskatG gene, a site-directed mutagenesis kit (GENEART^®^ Site-Directed Mutagenesis System, Invitrogen, Carlsbad, CA, USA) was used following the provided protocol. The process involved using two complementary oligonucleotides with the desired mutation in the center of their sequence (Supplementary Table S1) and was carried out through a polymerase chain reaction (PCR)-based method. Produced mutants were transformed in *E. coli* BL21 (DE3) electrocompetent cells (Merck, Kenilworth, NJ, USA) via electroporation on the BioRad Gene Pulser electroporator. The conditions used were 25 µF, 200 Ω, 1.5 V in 0.1 cm cuvettes (Sigma-Aldrich, Burlington, MA, USA). The selection of transformants was done on LB plates with ampicillin at 100 mg/L. The correct integration of plasmid and validity of mutations of the selected transformants was verified through sequencing, which employed specific CthedisKatG1 primers listed in Supplementary Table S1.

### 2.3. Heterologous Expression, Protein Purification, and Quantification

Recombinant CthediskatG and MagKatG1 were expressed in *E. coli* strain BL21 DE3 Star (Invitrogen) with addition of hemin (Serva, Heidelberg, Germany) (final conc. of 10 µM). Isopropyl β-D-thiogalactoside (IPTG, Sigma-Aldrich Burlington, MA, USA) (final conc. of 100 mM) was used as an inducer in M9ZL medium under conditions as described before with minor changes [5,11]. Expression was carried over night (for approx. 18 h) at 20 °C and cells were centrifuged for 15 min at 4500× *g* at 8 °C. Pellets were resuspended in homogenization buffer containing 50 mM sodium phosphate, pH 8.0, and 150 mM NaCl. Homogenization was done in three ultrasonication cycles on a Soniprep 150 Plus (MSE, Birmingham, UK) sonicator with the length of each cycle being 50 s, pulser set to 10.5, and intensive cooling for 2 min between each cycle. Crude homogenate was centrifuged for 20 min at 20,000× *g*, and soluble protein was concentrated using ammonium sulfate [12]. Crude concentrated homogenate of His-Tagged recombinant protein was then purified using IMAC with Ni-Sepharose Fast Flow (FF) affinity resin (Sigma Aldrich). Sodium phosphate buffer (0.1 M pH8.0) with imidazole (0.2–0.3 M) was used as an elution buffer. To remove imidazole from the buffer, a subsequent desalting was performed on PD-10 column (GE Healthcare, Chicago, IL, USA) containing Sephadex 20 resin. The final purification step involved gel filtration at 6 °C using a Superdex 200 (Increase 10/300 GL, Cytiva, Marlborough, MA, USA) column with a flowthrough of 0.5 mL/min. Selected fractions were pooled and concentrated up to 10 mg/mL using Centriprep 50 k (Merck, Kenilworth, NJ, USA) in 100 mM phosphate buffer, pH 8.0, NaCl 100 mM, and used for further analysis.

To confirm the size and purity of fractions, we used SDS-PAGE and native PAGE 4–15% gels from BioRad as well as Semi-dry Western blotting (Cytiva Amersham™ TE 70 PWR). All gels were stained with Coomassie Brilliant blue R-250 (Serva). Anti-HisTag (Penta-His™, Qiagen, Venlo, Netherlands) was used as primary and Antimouse IgG–AP (Sigma Aldrich) as secondary antibody for Western blot analysis. Nitrocelulose membrane was developed using alkaline phosphatase solution with BCIP/NBT Color Development Substrate (Promega, Fichtburg, WI, USA) allowing a color reaction with the bound antibody.

All concentration measurements were conducted using a NanoDrop 2000c spectrophotometer (Thermo Fisher Scientific, Waltham, MA, USA; Software ver. 1.4.2).

### 2.4. Nano-Differential Scanning Fluorimetry

Thermal denaturation of MagKatG1 and CthediskatG was monitored using nanoscale differential scanning fluorimetry (nanoDSF) measuring the 350-to-330 nm ratio changes of the intrinsic tryptophan and tyrosine fluorescence upon excitation at 280 nm in real time [13]. NanoDSF measurements were performed using Prometheus NT.48 equipment (NanoTemper Technologies, Munich, Germany) at protein concentrations in the 0.4–0.8 mg/mL range as determined by nanoDrop using the extinction coefficients 149,310 (MagKatG1), and 165,810 (CthediskatG) M^−1^ cm^−1^ at 280 nm. Temperature profiles were recorded from 20 to 95 °C with a ramping speed of 1 °C per minute. Measurements were repeated at least two times.

### 2.5. Electronic Circular Dichroism Spectroscopy

Electronic circular dichroism (ECD) spectra of highly purified catalase–peroxidase were recorded on Chirascan spectrophotometer (Applied Photophysics, Leatherhead, UK) flushed with nitrogen with a flow rate of 5 L/min. The following parameters were used for recording far-UV spectra: spectral range 260–190 nm, path length of 1 mm, spectral bandwidth 3 nm, step size 1 nm, and scan time 10 s per point. Thermal unfolding was monitored by stepwise increasing the temperature in the range 20–80 °C with the same spectral bandwidth and scan time. Recorded spectra were processed using Pro-Data Viewer software from Applied Photophysics (Version 4.1.9) and the content of secondary structure was deconvoluted with CDNN software.

### 2.6. Mass Spectrometry Analysis

First, 30 µg of isolated recombinant MagKatG1 or CthedisKatG proteins were diluted in 100 mM TrisHCl buffer, pH 7.8, and incubated briefly with trace amounts of hydrogen peroxide at 25 °C or 50 °C, respectively. Afterwards, they were reduced by 5 mM dithiothreitol and alkylated with 30 mM iodoacetamide. The alkylation reaction was quenched by an additional 5 mM dithiothreitol and the proteins were digested overnight using sequencing grade trypsin (1:20 *w*/*w*, Promega). Acidified (0.5% trifluoroacetic acid (TFA)) peptide solution was clarified by centrifugation, purified on a microtip C18 SPE, and dried. The peptides were dissolved in 0.1% TFA and 2% ACN, loaded (500 ng per run) onto a trap column (PepMap100 C18, 300 µm × 5 mm, Dionex, CA, USA), and separated with an Acclaim PepMap C18 column (75 µm × 500 mm, Thermo Scientific, Waltham, MA, USA) on Ultimate 3000 RSLCnano system (Dionex) in a 60-min gradient (3–43% B), curve 7, and flow-rate 300 nL/min. The two mobile phases were used: 0.1% formic acid (*v*/*v*) (A) and 80% ACN (*v*/*v*) with 0.1% formic acid (B). Eluted peptides were sprayed directly into Orbitrap Elite mass spectrometer (Thermo Scientific, Waltham, MA, USA) and spectral datasets were collected in the data-dependent mode using Top15 strategy for the selection of precursor ions for the HCD fragmentation [14]. The sample was analyzed in two technical replicates. Obtained datasets were processed by MaxQuant, version 1.5.3.30 [15] with built-in Andromeda search engine using carbamidomethylation (C) as permanent and oxidation (M) as variable modifications. The search was performed against *E. coli* protein database (UP000000625) and theoretical recombinant MagKatG1 and CthedisKatG1 amino acid sequences. The list of identified protein groups is presented in the Supplementary Table S2.

### 2.7. Enzyme Kinetics

Spectrophotometric measurements were used to determine the peroxidatic activity with various substrates. The reaction solution consisted of 10 mM peroxyacetic acid (Central chem) as electron acceptor with peroxidic bond. Substrates that served as electron donors were provided in the following concentrations of stock solutions: 3 mM ABTS (Sigma) (2,2′-azino-bis-3-ethylbenzothiazoline-6-sulfonic acid, ε_414_ = 31.1 mM^−1^ cm^−1^) [16]; 10 mM guaiacol (Sigma) (ε_470_ = 26.6 mM^−1^ cm^−1^) [17]; 12,5 mM L-DOPA (Merck) (L-Dihydorhyphenylalanine, ε_470_ = 3.6 mM^−1^ cm^−1^) [18]. For assessing the oxidation of manganese as an electron donor, 1 mM MnSO_4_ (ε_238_ = 6.5 mM^−1^ cm^−1^) [19] was used in 0.1 M potassium bitartrate buffer at pH 5.3. The temperature was varied stepwise from 20 °C to 70 °C while conducting measurements under two different pH conditions of the phosphate buffer, namely pH 5.8 and pH 7.0.

Catalatic activity was recorded as a decrease in the absorbance at 240 nm for hydrogen peroxide solution in 3 mL quartz cuvettes. Reaction mixture consisted of 100 mM phosphate buffer at pH 6.0 with 10 mM H_2_O_2_ (ε_240_ = 43.6 M^−1^ cm^−1^) [20]. Measurements were taken stepwise in temperature range from 20 °C to 70 °C.

All kinetic profiles of affinity purified enzymes were measured with Cary60 UV–Vis spectrophotometer (Agilent, Santa Clara, CA, USA) at constant temperatures using four chromogenous substrates for peroxidase activity or hydrogen peroxide for catalase activity.

### 2.8. Protein Structure Modeling and Ligand Transport Analysis

The sequence of CthedisKatG was used as an input for the BLAST search against the UniProt database. Uniprot sequence G0SFS6 (for CtheKatG) was identified as the closest homolog with 97% sequence identity. The 3D model of G0SFS6 was obtained from the AlphaFill database [21] and the structure of CthedisKatG was modeled using AlphaFold 2.0 [22]. The model structures of CthedisKatG and its closest homolog were superimposed and the prosthetic heme group was transplanted into the CthedisKatG active site. The 3D model of MagKatG1 was obtained directly from AlphaFill using the UniProt ID A4R5S9. Furthermore, both models were superimposed on the crystal structure of CAT-2 (PDB code 5 whs), and the covalent link between amino acids Met-Tyr-Trp was transplanted from the crystal to the model structures as AlphaFold is unable to capture important post-translational modifications. Finally, both models were further optimized using the 3DRefine server [23]. Secondary structure elements for investigated proteins were predicted with Jpred4 server [24].

The model structure of CthedisKatG was used as an input for the CaverWeb software [25] and prosthetic heme group was selected as a starting point of tunnel search. The heme group was preserved in the protein structure and the minimum probe radius was set to 0.9 A. Only four tunnels were detected by Caver [26], tunnel 1 being the most probable with bottleneck radius of 1.3 A and the total length of 11.9 A. Lower-bound ligand trajectories for guaiacol and L-DOPA were calculated using CaverDock software [27] in IN direction with the discretization radius set for 0.3 A.

## 3. Results

The investigation of fungal bifunctional catalase–peroxidases is first focused on potential differences in their sequences and structures, followed by detection of an essential post-translational modification in the active center and accomplished by comparison of recorded enzymatic activities of wild type and produced mutants.

### 3.1. Insight in the Peculiar Structure of Bifunctional Catalase–Peroxidases

Bifunctional catalase–peroxidases (E.C. 1.11.1.21) represent unique oxidoreductases that can offer an interesting variability of reaction mechanisms in the same active centers. Whereas the bacterial [28] and archaeal representatives have already been investigated for a long time [29], the eukaryotic representatives are less known. Among eukaryotic KatGs, the most abundant are fungal variants [9]. We can divide fungal catalase–peroxidases in mesophilic and thermophilic representatives dependent on their biological origin and particular habitat of their source [30]. Mainly, KatGs from thermophilic fungi are still not well documented but they can possess a potential for important biotechnological application due to their favorable thermostability. No crystal structure of a thermophilic KatG is known and attempts to crystallize it have so far failed. We therefore prepared models of a thermophilic catalase–peroxidase from *Chaetomium thermophilum* var. *dissitum* and a mesophilic catalase–peroxidase from *Magnaporte oryzae* using AlphaFold [22] and analyzed their structural properties.

**Figure 1:**
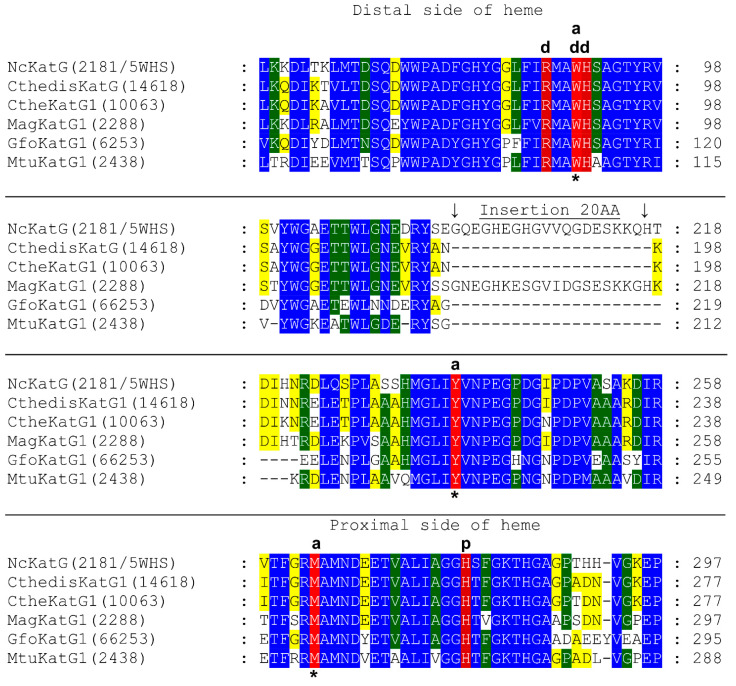
Conserved sections of the multiple sequence alignment involving chosen KatG protein sequences (each with corresponding PeroxiBase code) in comparison to the nearest identified experimental 3D crystal structure of a fungal catalase–peroxidase (PeroxiBase code 2181 or PDB code 5WHS, respectively) representing a catalase–peroxidase from *Neurospora crassa*). The conserved catalytic triad on the distal side of heme is marked with the letter d and the proximal heme side with the letter p. The abbreviations of listed sequences used correspond with PeroxiBase. Fungal representatives are also from Cthedis—*Chaetomium thermophilum* var. *dissitum*, Cthe—*Chaetomium thermophilum* var. *thermophilum*, and Mag—*Magnaporthe oryzae*. For comparison, two typical prokaryotic sequences are also presented below: Gfo—bacterium *Gramella forsetii*, Mtu—*Mycobacterium tuberculosis*. Color scheme: blue > 90%, green > 75%, yellow > 50% overall conservation, red—amino acids forming the covalent adduct also labeled also with an asterisk, dark red—essential amino acid residues on the distal and proximal side of heme. Numbers on the right correspond with the positions of the presented sequence in the whole amino acid sequence of a particular KatG.

In Figure 1, important aligned parts on the distal and proximal side of the prosthetic heme group can be seen for fungal and also related bacterial catalase–peroxidases. On the distal side of heme, the catalytic triad is formed by amino acid residues Arg87, Trp90, and His91 (numbering in CthedisKatG). Trp 90 appears essential for the catalase reaction mode but not for the peroxidase pathway as it is mutated in most evolutionary descendants lacking the catalase activity. It appears that behind this conserved catalytic triad, a large insertion of 20 amino acids occurs only in non-thermophilic KatG representatives in comparison with thermophilic counterparts represented by CthedisKatG and also by CtheKatG. Interestingly, this large insertion is also not present among bacterial catalase–peroxidases represented by sequences from GfoKatG1 and MtuKatG. This relatively large insertion can have an impact on the overall thermostability for the entire protein.

**Figure 2:**
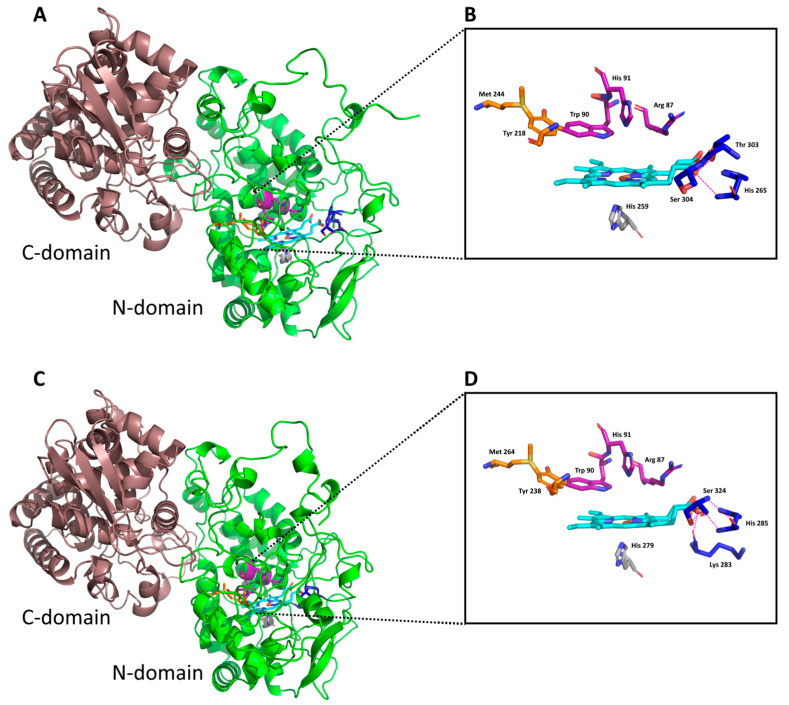
A 3D model of the CthedisKatG (**A**) and MagKatG1 (**C**) structure. Prediction of the monomeric structures of the proteins with two similar domains (N-domain in green and C-domain in red) are shown. The heme environment with distal and proximal heme site residues are shown as colored sticks and heme is highlighted with yellow stick color. The essential covalent adduct is formed by residues MYW: (**B**) Met244-Tyr218-Trp90 for CthedisKatG; (**D**) Met264-Tyr238-Trp90 for MagKatG1. The models were obtained with AlphaFold and rendered in PyMOL 2.5.4.

Models of monomer subunits of thermophilic CthedisKatG and mesophilic MagKatG1 bifunctional catalase–peroxidases show that both enzymes are organized in the same way (Figure 2). They are structurally similar to the crystal structure of the mezophillic CAT-2 from *Neurospora crassa,* with RMSDs of 0.68 Å and 0.74 Å for CthedisKatG and MagKatG1, respectively. Both enzymes are composed of two α-helical domains, an N-terminal (residues 1–448 and 1–428 for MagKatG1 and CthedisKatG, respectively) and a C-terminal (residues 449–750 and 429–723 for MagKatG1 and CthedisKatG, respectively; Figure 2). The packing of both domains is tight with the domain interface formed predominantly by loops from both domains and an α-helix from the C-terminal domain containing residues 605–616. The interdomain packing is strengthened by the insertion of the C-terminal domain loop, residues 491–497, into the N-terminal domain interface cavity. A charge–charge interaction between Asp448 and Arg497 in MagKatG1 (Asp428-Arg477 in CthedisKatG) likely has an important role in strengthening this interface. The main difference between the MagKatG1 and CthedisKatG structures is a 20-residue insertion (197–221) in the N-domain of MagKatG1 which forms a loop and a two-turn α-helix (209–215). This insertion is also present in other non-thermophilic KatG representatives (Figure 1).

**Figure 3:**
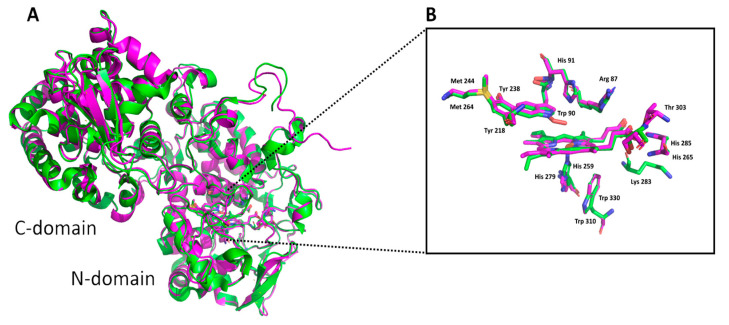
(**A**) Monomeric model structures of CthedisKatG (magenta) aligned with the crystal structure of CAT-2 (PDB code: 5WHS—green). Both enzymes have similar conformations with heme located in the N-terminal domain. The distal side of heme cavity (**B**) shows Arg-87, His-91, and Trp-90 involved in reaction with substrates. Proximal to the heme are His-259, Trp330 of CthedisKatG, and His-279 of CAT-2, respectively. The residues of the M-Y-W adduct are shown at the end of the entrance tunnel and on the opposite side are polar contacts of water molecules and residues with heme propionate (dotted lines). The models were obtained by homology modeling using AlphaFold [21] and rendered in PyMOL 2.5.4.

As is obvious from Figure 2, the structure of a thermostable KatG monomer composed of two domains is formed predominantly with α-helices. In analogy with all other already known catalase–peroxidases, the prosthetic heme group is at the bottom of a deep, centrally located cavity in the N-domain. Comparison of thermophilic CthedisKatG with the crystal structure of the mesophilic CAT-2 from *Neurospora crassa* (Figure 3) revealed no major differences in domain organization or the location of the active site. Based on this structural comparison and on an amino-acid sequence alignment with other eukaryotic KatGs (Figure 1), the catalytic site is likely formed by the Met244-Tyr218-Trp90 triad in CthedisKatG (Figure 2B) and the Met264-Tyr238-Trp90 triad in MagKatG1 (Figure 2D). The C-terminal domain does not bind heme, but it contributes to the overall fold and stability of entire KatG [5,9]. In Figure 2, two distinct fungal KatGs, namely thermophilic CthedisKatG and mesophilic MagKatG1, are compared. No obvious difference in the domain contents and organization is visible, and the active center appears to be highly conserved. Therefore, the peculiar details of differences between mesophilic and thermophilic catalase–peroxidases can be refined predominantly in the multiple sequence alignment of corresponding amino acid sequences.

**Figure 4:**
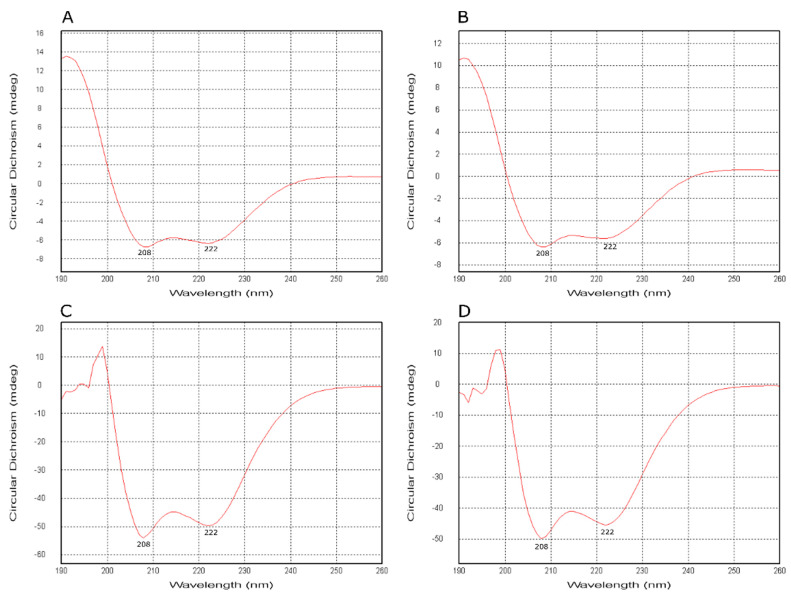
CD spectra of highly purified MagKatG1 and CthedisKatG (details in Section 2). Spectra were recorded at 20 °C for MagKatG1 and CthedisKatG ((**A**) and (**C**), respectively) and at 40 °C ((**B**) and (**D**), respectively). Both samples were diluted in 50 mM phosphate buffer pH 7.0 to the same final concentration. These far-UV spectra reveal mainly α-helical structural elements with a typical valley at 208 nm and a shoulder around 222 nm.

To verify the proposed structural models of both mesophilic and thermophilic KatGs, we monitored the content of secondary structure elements in affinity purified samples of MagKatG1 and CthedisKatG (Figure 4) via measurement of circular dichroism (CD spectrum) in the far UV region on the Chirascan device at two different temperatures. As can be observed in the output presented in Figure 4, both proteins contain a comparable portion of α-helices. However, this content is slightly higher in the thermostable variant because the corresponding ratio of signal intensity between 208 nm and 222 nm is higher for CthedisKatG at both observed temperatures. This output was further compared with prediction of secondary structure elements obtained from Jpred 4 server [24]. Results are summarized in Table 1. In the smaller thermostable CthedisKatG, the content of α-helices is almost 3% higher if compared with MagKatG1 and the content of β-strand is slightly lower. This can be explained partially by the presence of insertions only in the mesophilic KatG as demonstrated in sequence alignment of Figure 1. These insertions probably do not contain α-helical structure.

### 3.2. Investigation of Typical Covalent Adduct in Active Center of Fungal KatGs

To investigate the conservation of the active site in fungal KatGs with respect to its proposed bifunctionality, we created two distinct point mutations in recombinant CthedisKatG gene as described in Section 2.2 to test the architecture of the active center for its modified reactivity. These were substitutions of W90, which represents one of the catalytic triad residues and also part of the covalent adduct, either to V or to F. Their exact occurrence was verified by DNA sequencing of obtained clones after the mutagenesis that also confirmed no other unexpected mutations in the whole recombinant CthedisKatG gene.

**Figure 5:**
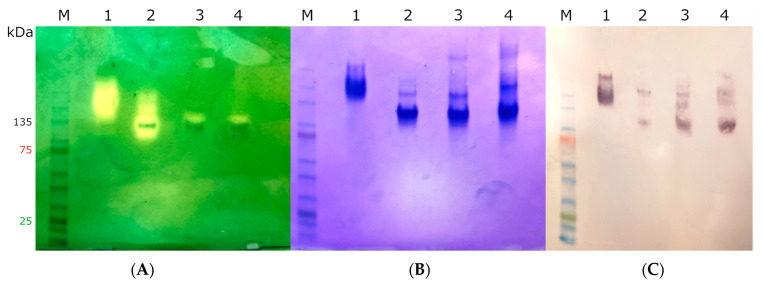
Native-PAGE (4–15%) and Western blot of (1) MagKatG1, (2) wild type CthedisKatG, (3) CthediskatG W90F, (4) CthedisKatG W90V mutants. (**A**) Qualitative catalase assay using staining with ferricyanide (III) and ferric chloride is shown on the left. (**B**) Coomassie stained Native PAGE is shown in the middle of the panel. (**C**) Corresponding developed nitrocellulose membrane with anti-His antibody. KatG proteins can be seen mainly in native dimeric form with calculated molecular weight of ≈160 kDa. Significant catalase activity can be seen in MagKatG1 (lane 1) and in the wild type CthedisKatG (lane 2) as yellow bands on a dark green background. With the introduction of site-directed mutants in the covalent M-Y-W adduct, the catalase activity was drastically reduced as obvious from lanes 3 and 4.

**Figure 6:**
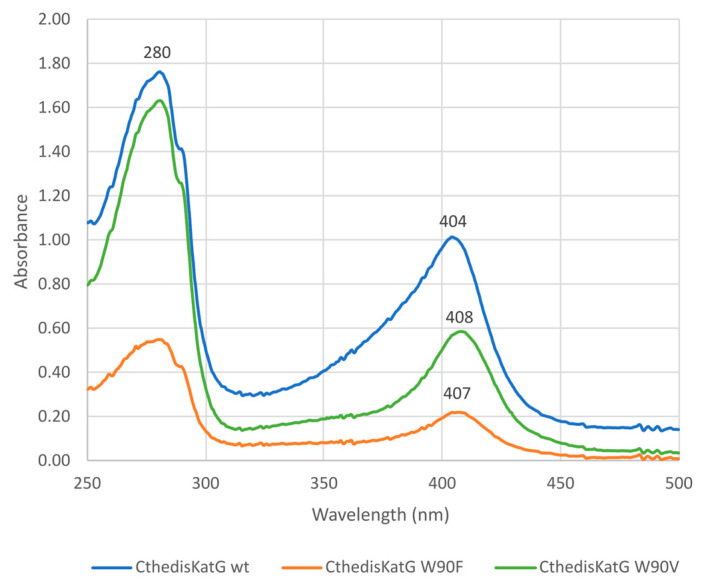
UV–vis spectra of affinity purified CthedisKatG (wild type) and of its two mutants (CthediskatG W90V and W90F) The ferric heme domain has a Soret maximum at around 404 nm. The purity ratio (RZ, A_404_/A_280_) value for the CthedisKatG was 0.57 and after the introduction of the point mutation in active site residues, values for CtG W90V and W90F mutants were 0.36 and 0.40, respectively. In contrast to wild type, mutants exhibited a slight shift in Soret band maximum (407 nm and 408 nm compared to 404 nm).

After purification of CthedisKatG—wild type and its two mutants CthedisKatG W90F and CthedisKatG W90V (Supplement S1)—we could demonstrate their different behavior in native protein electrophoresis (Figure 5). The catalase activity responsible for the typical staining was significantly lost in both mutant samples loaded on this gel in approximately the same concentration as for the wild type. This was demonstrated by staining of the identical gel with Coomassie brilliant blue. It can be concluded that mutant protein variants are produced in approximately the same amounts as the wild type, but their reactivity is significantly modified. To address this difference, we also investigated the UV–vis spectrum for highly purified samples of wild type and both produced mutants as shown in Figure 6.

The comparison of obtained electronic spectra reveals only small differences between them. The Soret maximum of both mutants is only slightly red-shifted in comparison to the wild type enzyme. However, as the purity ratio RZ is lower in both produced mutants, we can deduce that the incorporation of prosthetic heme group inside catalase–peroxidase is affected to a low extent in both mutants.

**Figure 7:**
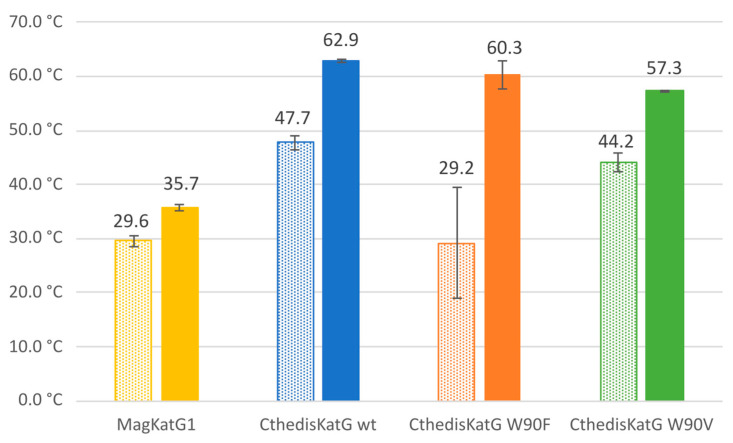
Quantitative determination of protein unfolding via measurement of intrinsic tryptophan fluorescence (Nano-DSF) for affinity purified MagKatG1, CthedisKatG wild type and for two point mutants. Data are presented as averages of first onset point (temperature at which unfolding begins—pattern columns) as well as inflection point (melting temperature or point at which 50% of protein is unfolded—solid fill columns) of studied enzymes measured on Prometheus NT.48. Data presented are averages ±SD.

The comparison of recorded changes in tryptophan fluorescence that reflects stepwise unfolding (Supplement S2) of investigated proteins [12] is shown in Figure 7. All presented samples of KatG were highly purified as demonstrated in corresponding protein gel electrophoresis (Figure 5) as well as by their RZ values (purity ratios) and distinct Soret peaks (Figure 6). NanoDSF results show a large difference in the thermal stability between wild-type MagKatG1 and thermophilic CthedisKatG (Figure 7); the melting point (Tm), where the 50% molecules are denaturated, for MagKatG1 is 35.7 °C vs. 62.9 °C for CthedisKatG. Likewise, the onset temperatures, where the molecules start to denaturate, are around 18 °C apart from each other with 29.6 and 47.7 °C for wild-type MagKatG1 and CthedisKatG, respectively. Both produced CthedisKatG mutants, W90F and W90V, show lower thermal stability with Tm of 60.3 and 57.3 °C, respectively, in comparison to CthedisKatG wt (Figure 7). A higher effect of W90V mutation on the CthedisKatG Tm is not surprising as tryptophan and phenylalanine are both aromatic amino-acids with more similar side-chain structure in comparison to valine.

**Figure 8:**
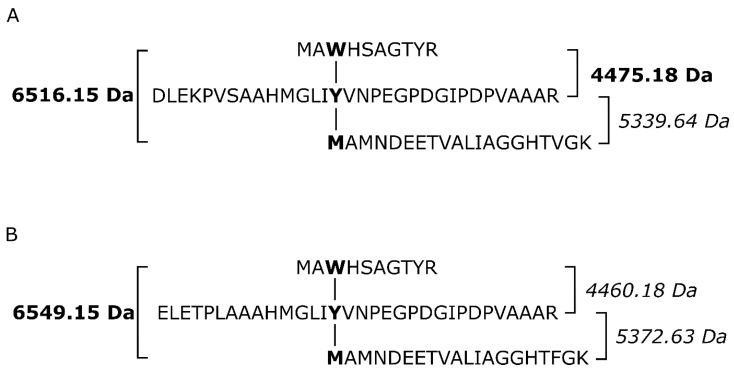
Schematic presentation of covalently linked peptides forming the typical post-translational modification responsible for a high catalase activity of KatGs. (**A**) involved peptides of MagKatG1 sequence, (**B**) involved peptides of CthedisKatG. Details of the analysis are shown in Supplementary Table S2.

It is known that an important post-translational modification known as a covalent adduct on the distal side of heme in all known catalase–peroxidases is responsible for their significant catalase activity [6,10] producing water and molecular oxygen. This specific modification is reflected in the presence of a spontaneous crosslinking between amino acids Met264-Tyr238-Trp90 for MagKatG1 and Met244-Tyr218-Trp90 for CthedisKatG (Figure 1 and Figure 3). Proteomic analysis of both isolated catalase–peroxidases revealed the presence of uncoupled peptides containing the mentioned unique adduct. However, such routine proteomic analysis does not allow the identification of covalently coupled peptides. In the crosslinked tripeptide search, we analyzed obtained LC-MS data using extracted ion chromatograms (XIC) for the calculated ion forms of the crosslinked targets (Supplementary Table S2). Monoisotopic molecular weights of all relevant peptide combinations were calculated by the mathematical sum of individual peptides, disregarding mass losses due to covalent bond formation. Coelution of m/z signals in XIC for combinations of several ion forms of target crosslinked peptide indicated its presence in the dataset. The mass spectrum of the corresponding time window showed the presence of expected sets of multiple charged m/z signals. Mathematical deconvolution led to the identification of the monoisotopic masses for mesophilic MagKatG1 in the peptides 1-2-3 of 6516 Da and for the thermophilic CthedisKatG, a similar value for analogous peptides 1-2-3 of 6549 Da. Additionally, for MagKatG1, the peptide combination 1-2 of 4475 Da was also detected. These values differed by 4 Da in the case of triple peptides and only by 2 Da in the case of the double peptide of MagKatG1 (Supplementary Table S2). The mass difference corresponds to the loss of two hydrogen atoms for each covalent bond created. This means that irrespective of their natural origin and physiological state, both fungal catalase–peroxidases contain a conserved post-translational modification on the distal heme side known as covalent adduct. A schematic view of detected covalently linked peptides is presented in Figure 8 for both enzymes. It was proposed that this essential PTM enables a high specific catalase activity in active center evolved for mainly peroxidase activity [8,9].

### 3.3. Comparison of Enzymatic Activities of Wild Type KatGs and Point Mutants

**Figure 9:**
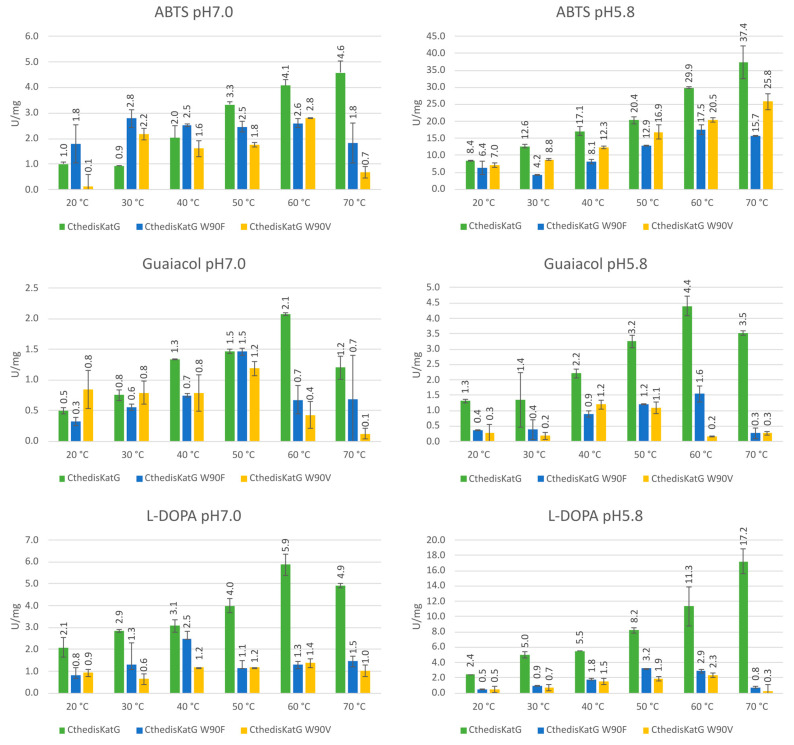
Graphical representation of specific peroxidase activities of the wild type CthediskatG as well as produced point mutants in the active center. Data are presented as unit of enzyme (U) per mg of protein ± SD.

The proposed bifunctionality of fungal catalase–peroxidases can easily be verified spectrophotometrically. Peroxidase activities were recorded for the thermostable enzyme CthedisKatG—wild type and for both produced point mutants with three typical substrates serving as common electron donors of most peroxidases. Results obtained at two different pH values are presented in Figure 9. For wild type, the highest obtained specific activity was achieved for ABTS at 70 °C but for guaiacol only at 60 °C. For L-DOPA, the obtained maximum of specific activity was dependent on the pH value of buffer but also 60 °C and 70 °C maxima were achieved. For both investigated point mutants, these specific activities were generally lower in all cases but most significantly with L-DOPA at elevated temperatures.

**Figure 10:**
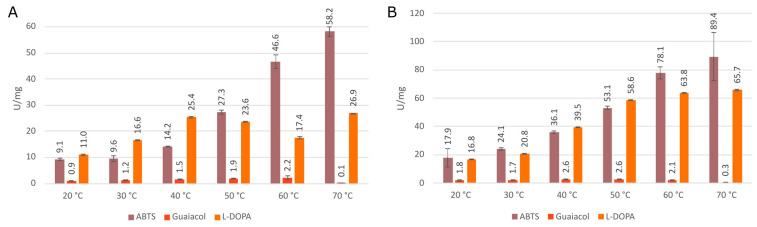
Specific peroxidase activities of MagKatG1 with various substrates and temperatures in pH7.0 (**A**) and pH5.8 (**B**). Data are presented as unit of enzyme (U) per mg of protein ± SD.

Further, peroxidase activities with these three typical substrates were also monitored for the mesophilic counterpart MagKatG1 and corresponding results are presented in Figure 10 for a direct comparison with the output from thermophilic variants. It is interesting to observe that although claimed only as mesophilic, MagKatG1 still has a rather high peroxidase activity with ABTS even at elevated temperatures of 60 °C and 70 °C. However, the activities with L-DOPA and mainly with guaiacol are much lower at higher temperatures.

**Figure 11:**
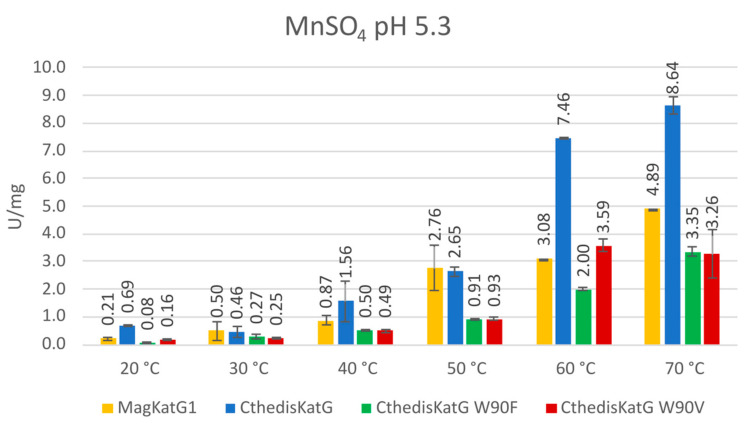
Specific manganese–peroxidase activity of MagKatG1, CthediskatG, and its two point mutants. Data are presented as units of enzyme (U) per mg of protein ± SD.

In addition to classical peroxidase substrates, we also tested the manganese peroxidase activities in all the here-presented peroxidase samples. This was performed spectrophotometrically by following the oxidation of Mn^2+^ to Mn^3+^ at 238 nm. It is already well known that Mn^3+^ as diffusible chelate is crucial for the oxidation of phenolic moiety in wood lignin performed by numerous fungi of various phyla and lifestyle [31]. Therefore, it is important to also test purified fungal KatGs of various origin for this potential ability. Obtained results are presented in Figure 11. It is interesting to see that manganese peroxidase activity increased significantly at elevated temperatures for all 4 tested KatG variants but it was highest for the wild type of CthedisKatG at 60 °C and 70 °C. At low temperatures, this type of activity does not appear to be significant for any investigated KatG. This means that mainly for phytopathogenic MagKatG1, it has no physiological significance because this property cannot be exploited by the phytopathogenic fungus *Magnaporthe oryzae* under 50 °C. However, CthedisKatG can probably react in this way at optimal growth temperature of this saprophytic fungus.

**Figure 12:**
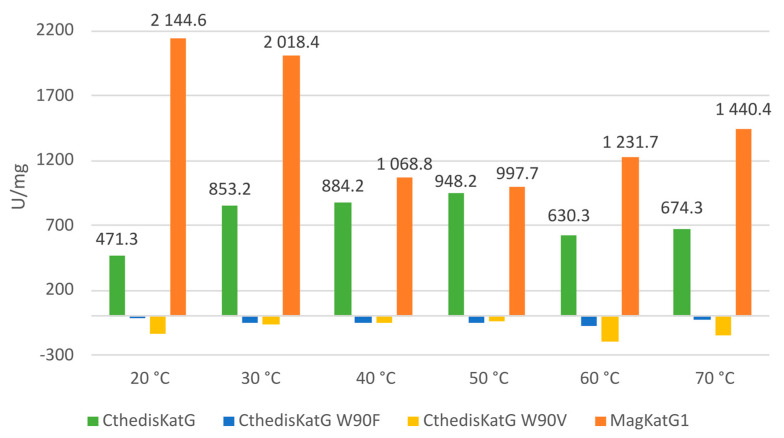
Catalase activity recorded for wild type CthediskatG, its two point mutants, and for MagKatG1. Data are presented as units of enzyme (U) per mg of purified protein.

Finally, we recorded the catalase activities for all the presented purified enzymes as the decomposition of hydrogen peroxide into water and molecular oxygen followed spectrophotometrically at 240 nm as decrease in the H_2_O_2_ concentration. The difference between natural enzymes and produced point mutants is obvious in Figure 12.

From recorded results, it is apparent that both produced mutants that destruct the covalent adduct present in all known catalase–peroxidases (Figure 1 and Figure 3) lead to a total loss of the catalase activity, i.e., the decomposition of hydrogen peroxide into water and molecular oxygen is not possible in them. Indeed, no detectable catalase activity could be recorded for both CthedisKatG variants represented by mutations W to F and W to V (Figure 12). However, the peroxidase activity shown in Figure 9 and Figure 11 as the oxidation of various electron donors (after activation of KatG with peroxide) remains relatively high but still lower if compared with the unmodified enzyme CthedisKatG.

### 3.4. Docking of Substrates in the Active Center of Bifunctional Catalase–Peroxidase

To elucidate the observed reactivity of wild type CthedisKatG and prepared point mutants with various peroxidase substrates, we used CaverDock to model optimal ligand binding trajectory from the surface of the protein to its active site.

**Figure 13:**
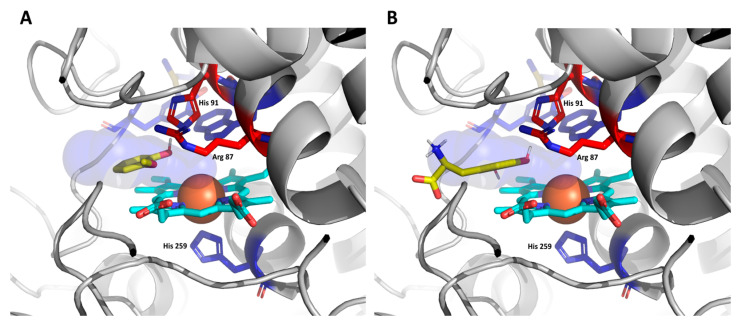
Analysis of potential binding of peroxidase substrates in the active center of wild type CthediskatG. (**A**) Docking of guaiacol and (**B**) docking of L-DOPA. Color scheme: green/yellow—substrates serving as electron donors, cyan—prosthetic heme group (with Fe^3+^ orange), red—essential amino acids on the distal side involved in the reaction mechanism. Blue—conserved amino acids in the active center of KatG.

Figure 13 captures the predicted binding site of guaiacol (A) and L-DOPA (B). From this figure, their respectable OH groups can be observed to interact directly with CthedisKatG active site represented by catalytic residues Arg 87, His 91, and the heme group. The binding energy of guaiacol during its oxidation ranges from 2.5 to 2.7 kcal/mol with the energy barrier (energy of the transport) between 6.2 and 6.4 kcal/mol. Similarly, the binding energy of L-DOPA is around the value of 0.8 kcal/mol with the energy barrier around 3.1 kcal/mol. The predicted energy barriers would suggest that both guaiacol and L-DOPA can be transported into the CthedisKatG active site. On the other hand, the positive binding energies show that structural flexibility is necessary to accommodate the ligands properly, which is not covered by CaverDock calculation. In contrast, the ABTS molecule is too large for the constraints defined by the protein tunnel and the heme cavity. Therefore, it cannot be positioned in the active center of CthedisKatG. It is expected that this special synthetic substrate is oxidized on the surface of the protein molecule via an electron transfer pathway between heme cofactor and specific amino acid residues of fungal KatGs in a similar way as resolved for bacterial catalase–peroxidases [32]. This hypothesis is supported by the fact that even a partially unfolded molecule of MagKatG1 (cf. Figure 7) still reveals a high activity with ABTS at elevated temperatures (Figure 10).

**Figure 14:**
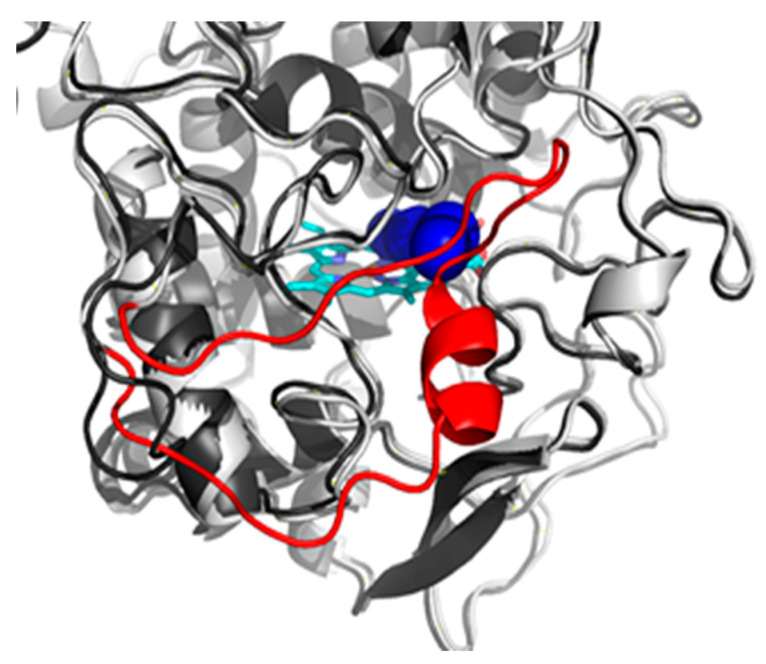
Model structure of the CthedisKatG (dark gray) aligned to the model structure of the MagKatG1 (light gray). The red color highlights the unique insertion of 20 amino acids to the sequence of MagKatG1 leading to the formation of an additional loop blocking the entrance of the main tunnel of CthedisKatG (blue), cyan - prosthetic heme group.

Compared to the thermophilic CthedisKatG, MagKatG1 contains a twenty-amino acid-long insertion in close proximity to the protein’s main tunnel opening. This insertion leads to the formation of an extra loop (Figure 14) preventing the ligands’ transport from the protein surface to its active site. While the main tunnel of the CthedisKatg is permanently opened, the inserted loop of MagKatG1 suggests some level of structural flexibility. Due to this reason, CaverDock analysis could not be carried out as the algorithm is limited to the static structure where the main tunnel is obstructed by the inserted loop.

The presented extra loop that also occurs in *Neurospora crassa* catalase–peroxidase (Figure 1) distinguishes fungal mesophilic KatGs from their thermophilic counterparts but apparently also from bacterial KatGs. Thermophilic fungal KatGs that are generally shorter reveal a higher unfolding stability (Figure 7) in comparison to mesophilic KatGs and also their activities are comparable. Thus, the role of this twenty-amino-acids long loop in fungal mesophilic catalase–peroxidases is unknown.

## 4. Discussion

Our research is focused on systematic analysis of the molecular diversity and reactivity of distinct peroxidase and catalase superfamilies with an outlook on their future applications in green technologies. Two applications can be mentioned here as typical examples. First, there is the possibility of efficient degradation of electron rich aromatic residues in waste waters, as they are among typical peroxidase substrates [33]. Second, a specifically developed method allows the removal of colored stamps from various types of papers [34] with the usage of CthedisKatG. Recently, we presented typical features of fungal hybrid B heme peroxidases [35,36] and also of fungal heme peroxygenases [37] that reveal a slightly different reaction mechanism to classical peroxidases. In contrast to all the above mentioned peroxidase families, bifunctional catalase–peroxidases possess, in addition to a highly variable peroxidase activity (comprising many different natural and synthetic substrates), a significant catalase activity. This means that bifunctional KatGs are also capable of a rapid decomposition of harmful hydrogen peroxide into water and molecular oxygen. This physiologically important reaction property is analogous to typical heme catalases that form another distinct protein family and do not oxidize most available peroxidase substrates. In this contribution, we try to demonstrate the typical architecture of the active center of bifunctional fungal KatGs, otherwise very similar to monofunctional heme peroxidases represented by, e.g., ascorbate- or lignin peroxidases. As there is no other detected reaction center in fungal KatGs, the one with heme located in the N-terminal domain must serve for both reaction possibilities. We detected the essential post-translational modification represented by a covalent adduct of three conserved amino acids on the distal side of heme for both thermophilic and mesophilic fungal KatG variants. Presented results obtained mainly from mass spectrometry analysis of highly purified KatGs detected covalently linked tripeptides of the same sequence part in both fungal KatG proteins. They reveal the presence of proposed post-translational modification in ascomycetous bifunctional KatGs. We have demonstrated that this essential covalent adduct occurs spontaneously after short incubation of folded KatG protein with trace amounts of hydrogen peroxide at temperatures that are optimal for each investigated enzyme. We can confirm that this PTM is located precisely in the same sequence motif as observed for cyanobacterial KatGs before [10]. Moreover, we purposively modified in the gene for fungal thermostable KatG, the DNA region coding for a part of highly conserved sequence motif coding for this covalent adduct. This was achieved through a single substitution leading to the exchange of the distal Trp to non-polar amino acid variants Phe or Val. After efficient purification protocol, we showed on the protein level that this KatG essential residue in position Trp90 (in both here investigated cases) is responsible for a high catalase activity as a part of the catalytic triad near the heme iron. This peroxide dismutation activity was fully removed after mutation of W to V or F. We showed further that through introduction of the mentioned point mutations in an otherwise highly conserved position on the distal side of the prosthetic heme group, peroxidase activities with various one electron donors were also negatively influenced to some extent in both engineered variants. Related heme peroxidases from the same protein superfamily that do not possess this PTM are not able to dismutate hydrogen peroxide efficiently although they possess a very similar architecture of the active center. They can only react in the peroxidase pathway by oxidizing various substrates with concomitant reduction of hydrogen peroxide to water as was shown for their various purified representatives, e.g., [31,35,36]. Moreover, the results recorded from monitored protein thermal unfolding demonstrate that after introduction of these single point mutations in (originally) thermostable KatG, the overall stability of both produced mutants was negatively influenced in comparison to the rather high folding stability of the wild type enzyme. The recorded denaturation profile for the thermostable CthedisKatG is unambiguously higher in comparison to the mesophilic MagKatG1. It is interesting to point out that according to the presented multiple sequence alignment (Figure 1), a relatively large insertion comprising 20 amino acids occurs between the distal and proximal side of prosthetic heme group only among mesophilic fungal catalase–peroxidases (visible in Figure 14). Although the thermostable variants appear to have a quite compact mostly α-helical structure, the specific enzymatic activity of mesophilic MagKatG1 is comparable with thermophilic CthedisKatG, even at elevated temperatures. This appears surprising but it needs to be mentioned that previous phylogenetic analysis revealed a phylogenetic tree where all fungal KatGs are proposed as evolutionary descendants of a single HGT event from certain bacterial donors (documented in [8,9]. Thus, the origin of KatG thermostability can probably be found in the very ancestral bacterial predecessors of the whole KatG protein family. In descendant fungal KatGs, it was probably preserved although not always needed. By summing up the obtained results, the introduction of point mutations in a fungal thermostable oxidoreductase in otherwise highly conserved protein region near the active site can not only modify the resulting enzyme activity but also lead to a modified protein with significantly reduced unfolding stability. This means that during preparation of directed mutations in genes coding for natural proteins in the perspective of certain application purposes (e.g., wastewater treatment or removal of industrial dyes), one has to be mindful of the impact of produced local mutations on the stability of the entire multidomain protein.

## 5. Conclusions

We produced two unique point mutations of a thermostable fungal catalase–peroxidase and compared their reactivity with unmodified thermostable enzyme as well as related enzyme from a mesophilic pathogenic fungus. These results underline the importance of a post-translational modification on the distal side of the prosthetic heme group known as a covalent adduct in bifunctional catalase–peroxidases formed with a spontaneous covalent link between three highly conserved amino acids of KatGs.

## Figures and Tables

**Table 1:** Contents of secondary structure elements in highly purified mesophilic and thermophilic catalase–peroxidases based on the prediction from Jpred 4 server [23].

Protein	MagKatG1	CthedisKatG
α-helix	29.6%	32.5%
β-strand	5.1%	4.6%
Mw subunit	83.476 kDa	80.819 kDa
ε_280nm_	165.81	149.31

## Data Availability

Sequence data for presented proteins are available at https://peroxibase.toulouse.inra.fr, (accessed on 28 June 2023). All obtained experimental data presented in this study are available on request from the corresponding author.

## Supplementary Materials

The following supporting information can be downloaded at: https://www.mdpi.com/article/10.3390/antiox12071382/s1, Supplementary Table S1—DNA Primers; Supplementary Table S2—KatG Xlinked peptides Mw_XIC; Supplement S1—CthedisKatG and MagKatG1 FPLC purification; Supplement S2—NanoDSF spectra of CthedisKatG and mutants.
