# Supplementary material for: Comparison of Fungal Thermophilic and Mesophilic Catalase–Peroxidases for Their Antioxidative Properties
Source: Antioxidants (Basel). 2023 Jul 4;12(7):1382. doi: 10.3390/antiox12071382 (PMC10376177; doi:10.3390/antiox12071382)
Supplement: Supplementary file 1 [file antioxidants-12-01382-s001.zip › Suplement S1 - CthedisKatG and MagKatG1 FPLC.pdf]

Proteins were purified by FPLC using Superdex200 10/300 GL column. Fractions from chromatography were pooled together based on chromatograph from FPLC.

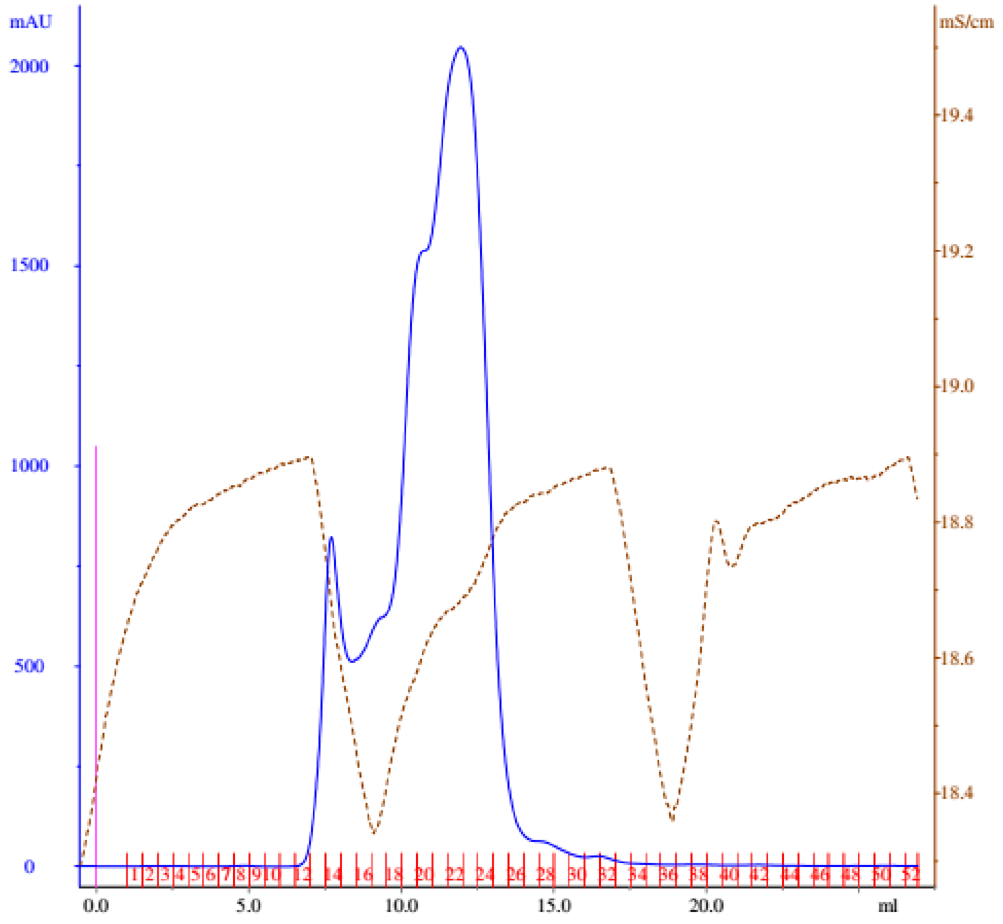

Figure S1. Chromatograph showing purification results of CthediskatG. UV captured at 280nm.

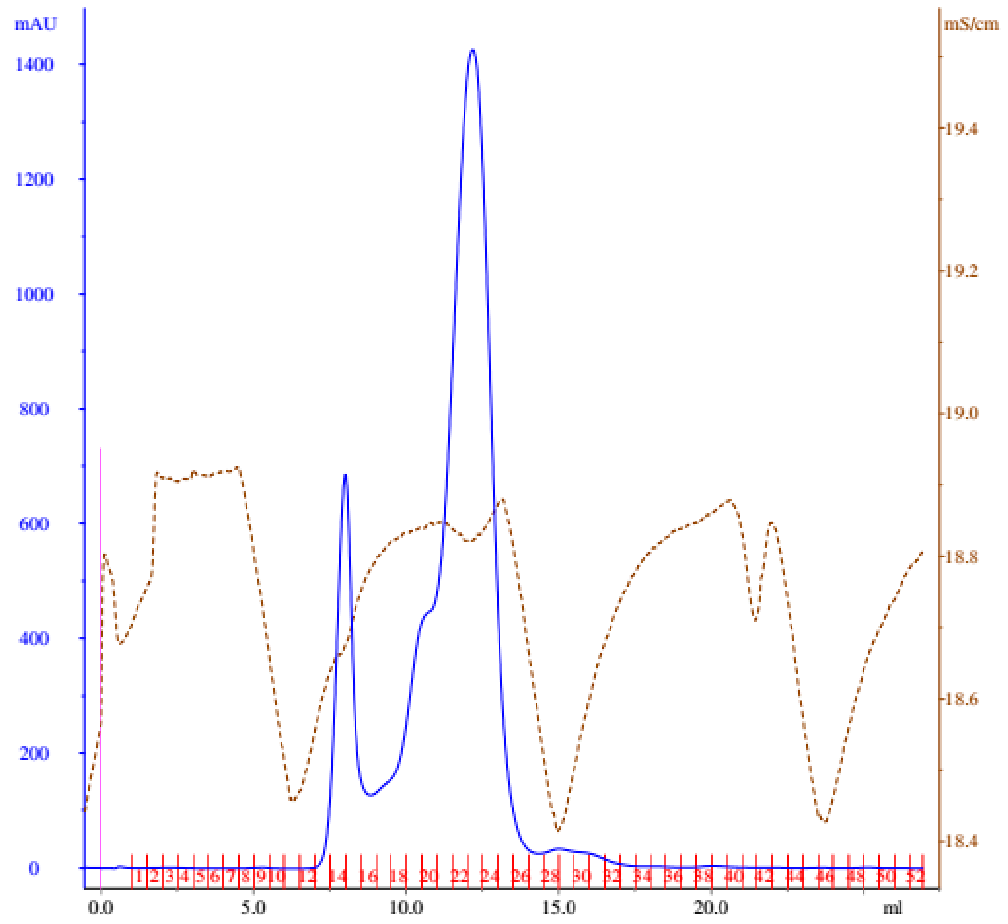

Figure S2. Chromatograph showing purification results of MagkatG1. UV captured at 280nm.

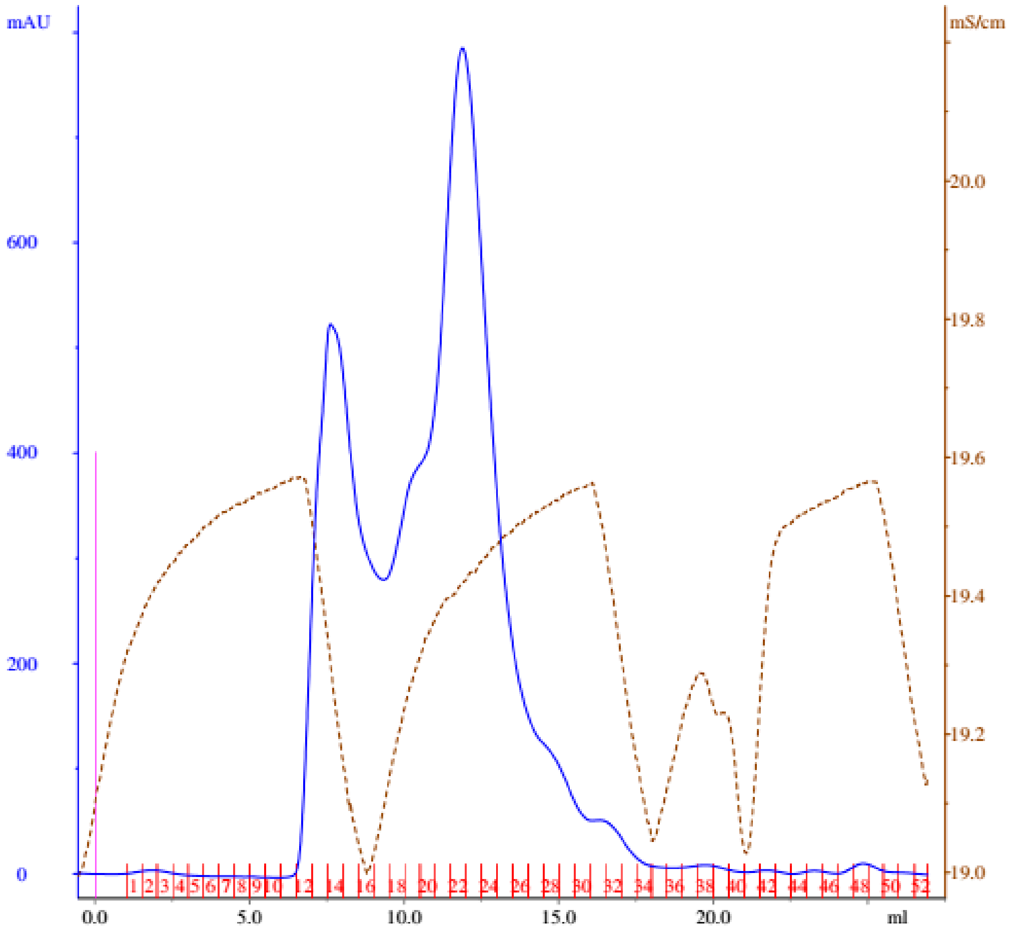

Figure S3. Chromatograph showing purification results of CthedisKatG W90V. UV captured at 280nm.

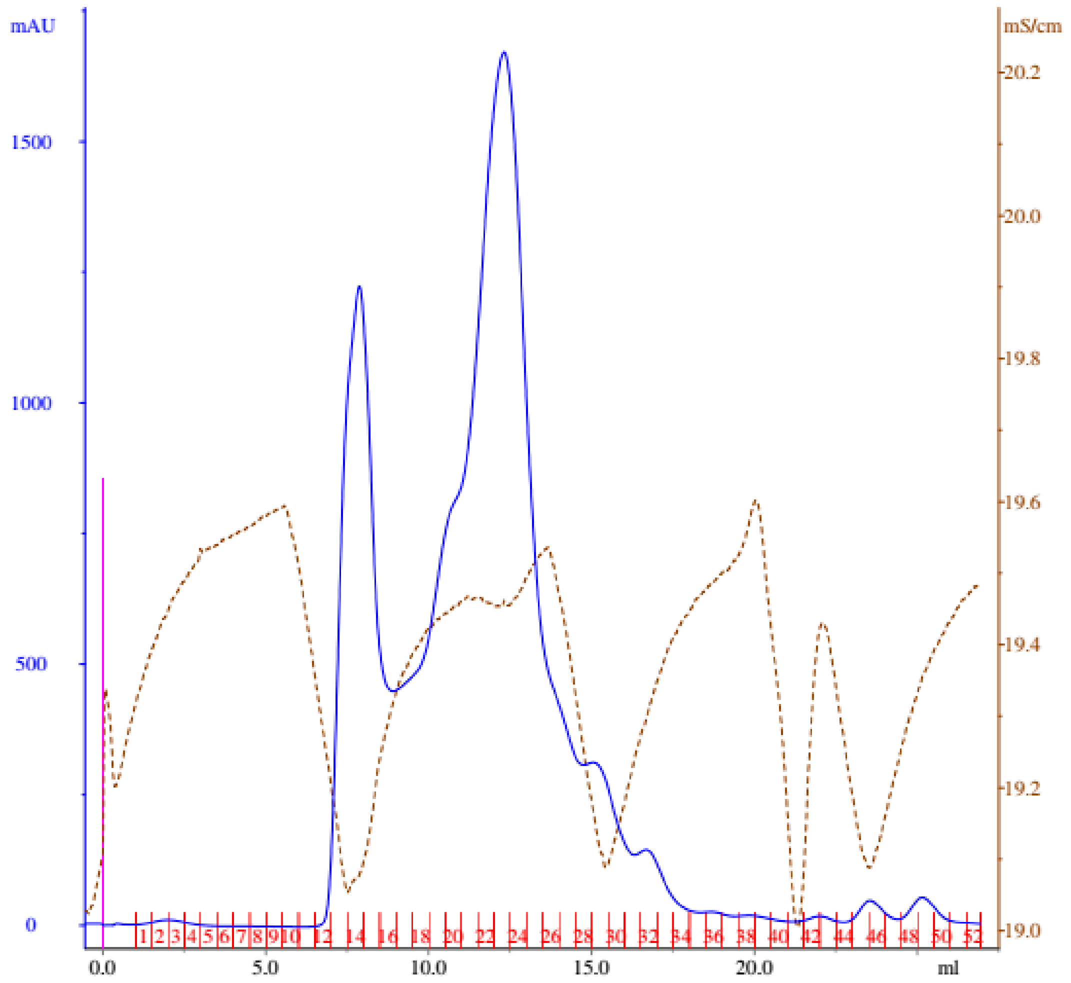

Figure S4. Chromatograph showing purification results of CthedisKatG W90F UV captured at 280nm.
