# Supplementary material for: Comparison of Fungal Thermophilic and Mesophilic Catalase–Peroxidases for Their Antioxidative Properties
Source: Antioxidants (Basel). 2023 Jul 4;12(7):1382. doi: 10.3390/antiox12071382 (PMC10376177; doi:10.3390/antiox12071382)
Supplement: Supplementary file 1 [file antioxidants-12-01382-s001.zip › Suplement S2 - NanoDSF spectra of CthedisKatG and mutants.pdf]

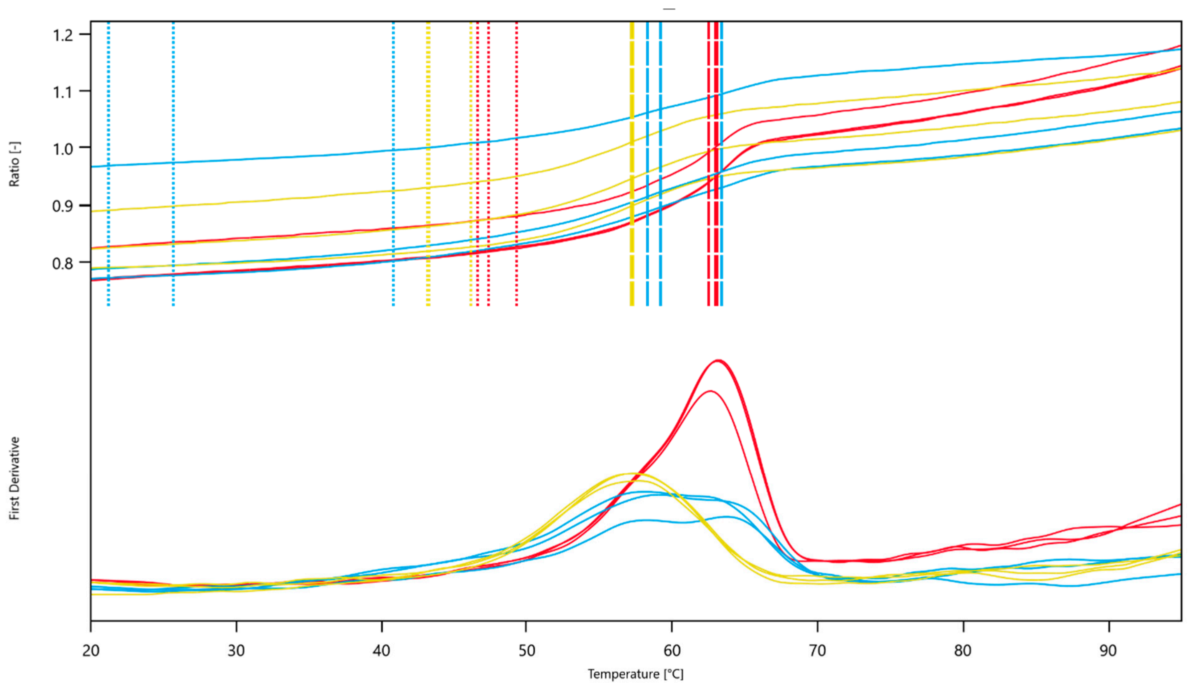

Figure S5. NanoDSF spectra thermal denaturation of the CthedisKatG and mutants. CthedisKatG wt is shown in red, CthedisKatG W90F in blue and CthedisKatG W90V in yellow.
