# Supplementary material for: Comparison of Fungal Thermophilic and Mesophilic Catalase–Peroxidases for Their Antioxidative Properties
Source: Antioxidants (Basel). 2023 Jul 4;12(7):1382. doi: 10.3390/antiox12071382 (PMC10376177; doi:10.3390/antiox12071382)
Supplement: Supplementary file 1 [file antioxidants-12-01382-s001.zip › Supplementary Table S1 - Primers.pdf]

Supplementary Table S1. Primers.

Gene	Primer description	Sequence in 5´→ 3´ direction	Tm [°C]
CthediskatG1 mutagenesis	W90F_V_FWD	TTTATTCGTATGGCAKTCCATAGCGCAGGCA	73
	W90F_V_REV	TGCCTGCGCTATGGAMTGCCATACGAATAAA	73
CthedisKatG1 sequencing	CtG.M2-Fwd	GCATTCAAAAGTCTGGATTATGAAGC	64
	CtG.M2-Rev	CTTCCGGATTAACATAGATCAGACC	64
	CtG.M1-Fwd	GCGAAATTAATACGACTCACTATAGG	62
	CtG.M1-Rev	CCAAGGGGTTATGCTAGTTATTGC	66
